# Cell Type-Specific Effects of Implant Provisional Restoration Materials on the Growth and Function of Human Fibroblasts and Osteoblasts

**DOI:** 10.3390/biomimetics7040243

**Published:** 2022-12-16

**Authors:** Takanori Matsuura, Keiji Komatsu, Denny Chao, Yu-Chun Lin, Nimish Oberoi, Kalie McCulloch, James Cheng, Daniela Orellana, Takahiro Ogawa

**Affiliations:** Division of Regenerative and Reconstructive Sciences and Weintraub Center for Reconstructive Biotechnology, UCLA School of Dentistry, Los Angeles, CA 90095-1668, USA

**Keywords:** peri-implant tissue, provisional restoration, fibroblast, osteoblast, cytotoxicity

## Abstract

Implant provisional restorations should ideally be nontoxic to the contacting and adjacent tissues, create anatomical and biophysiological stability, and establish a soft tissue seal through interactions between prosthesis, soft tissue, and alveolar bone. However, there is a lack of robust, systematic, and fundamental data to inform clinical decision making. Here we systematically explored the biocompatibility of fibroblasts and osteoblasts in direct contact with, or close proximity to, provisional restoration materials. Human gingival fibroblasts and osteoblasts were cultured on the “contact” effect and around the “proximity” effect with various provisional materials: bis-acrylic, composite, self-curing acrylic, and milled acrylic, with titanium alloy as a bioinert control. The number of fibroblasts and osteoblasts surviving and attaching to and around the materials varied considerably depending on the material, with milled acrylic the most biocompatible and similar to titanium alloy, followed by self-curing acrylic and little to no attachment on or around bis-acrylic and composite materials. Milled and self-curing acrylics similarly favored subsequent cellular proliferation and physiological functions such as collagen production in fibroblasts and alkaline phosphatase activity in osteoblasts. Neither fibroblasts nor osteoblasts showed a functional phenotype when cultured with bis-acrylic or composite. By calculating a biocompatibility index for each material, we established that fibroblasts were more resistant to the cytotoxicity induced by most materials in direct contact, however, the osteoblasts were more resistant when the materials were in close proximity. In conclusion, there was a wide variation in the cytotoxicity of implant provisional restoration materials ranging from lethal and tolerant to near inert, and this cytotoxicity may be received differently between the different cell types and depending on their physical interrelationships.

## 1. Introduction

Prosthetic restorations using dental implants have increased in popularity and prevalence [1]. However, the occurrence of peri-implant complications such as peri-implant mucositis and peri-implantitis is rapidly increasing [2]. For dental implant restoration, the prosthesis must extend from the implant platform through the connective tissue and junctional epithelium into the oral cavity. Depending on the implant position, tissue healing, and prosthetic strategy, the alveolar bone can be in close proximity to provisional restorations. Ideally, the materials used for this transmucosal portion should be nontoxic to the contacting and adjacent tissues and should not induce peri-implant gingival recession and bone loss [3,4,5,6]. Preferably, the materials should allow peri-implant connective tissue to adhere and form a stable physical barrier to prevent the invasion of oral bacteria, which could jeopardize osseointegration and implant longevity [4,7]. Thus, prior to delivery of the definitive implant restoration, provisional implant restorations play important roles in establishing anatomical and biophysiological stability and a soft tissue seal during the critical stage of healing [8,9].

Various materials such as self-curing poly (methyl methacrylate) (PMMA) acrylics, milled PMMA acrylics, bis-acrylics, and composite resins are used in implant provisional restorations [10,11,12]. Self-curing acrylics are used on demand [13,14,15], while milled PMMA acrylics are made from a PMMA block/disc prepolymerized at a high temperature and pressure and subsequently machined using computer-aided design/computer-aided manufacturing (CAD/CAM) systems. Unlike PMMA, bis-acrylics contain filler particles and shrink less after polymerization. Composite resins are made of inorganic fillers, photoinitiators, and matrix monomers such as bisphenol A glycidyl methacrylate (bis-GMA) and urethane dimethacrylate (UDMA).

The chemical composition of polymer-based materials might alter the biological properties and responses of the peri-implant soft tissue. A number of studies have reported the cytotoxicity of acrylics, which is variable due to the chemical composition, type, and quantity of the leaching residual monomer [16,17,18,19,20,21,22,23,24,25,26,27,28,29,30]. Polymer-based materials also generate free radicals during and after polymerization, which cause significant cellular damage [19,20,26,31]. Conversely, prefabricated PMMA blocks are assumed to produce minimal or no residual monomers or free radicals. Despite emerging theoretical knowledge about the properties and effects of these materials, the choice of material remains largely based on operator preference with limited consideration of their biocompatibility, most likely due to a lack of robust, systematic, and fundamental data to inform clinical decision-making.

While there have been several studies of cellular responses to definitive implant restoration materials, there is less data on the commonly used provisional restoration materials [12,16,32,33]. More importantly, a very limited number of studies have examined the effects of provisional materials on multiple cell types simultaneously [34]. This is important since the biocompatibility of provisional materials is likely to influence both soft and hard tissue cells in different locations, so care must also be taken to consider the positional relationship between the cells and material to mimic the clinical context. Therefore, the objectives of this study were to (i) evaluate the behavior and function of human oral fibroblasts and osteoblasts in the presence of five different provisional dental restoration materials including titanium (Ti) alloy as a bioinert control, and (ii) compare responses of the two different cell types to determine potential material-cell type crossover modulation. Cellular behavior and function were separately examined for cells in direct contact with, and in close proximity to, the materials to mimic the intraoral environment.

We hypothesize that the biocompatibility of implant provisional materials varies more significantly than we anticipate in daily clinical practice and that the adverse effect of selected materials remains significant even in proximity, without direct contact. We also postulate that osteoblasts, which are categorized into differentiating cells, are more susceptible to material toxicity than nondifferentiating gingival fibroblasts. The would-be-obtained results in this study will provide a foundation for understanding and selecting various materials during implant provisional restoration.

## 2. Materials and Methods

### 2.1. Material Preparation and Characterization

**Figure 1:**
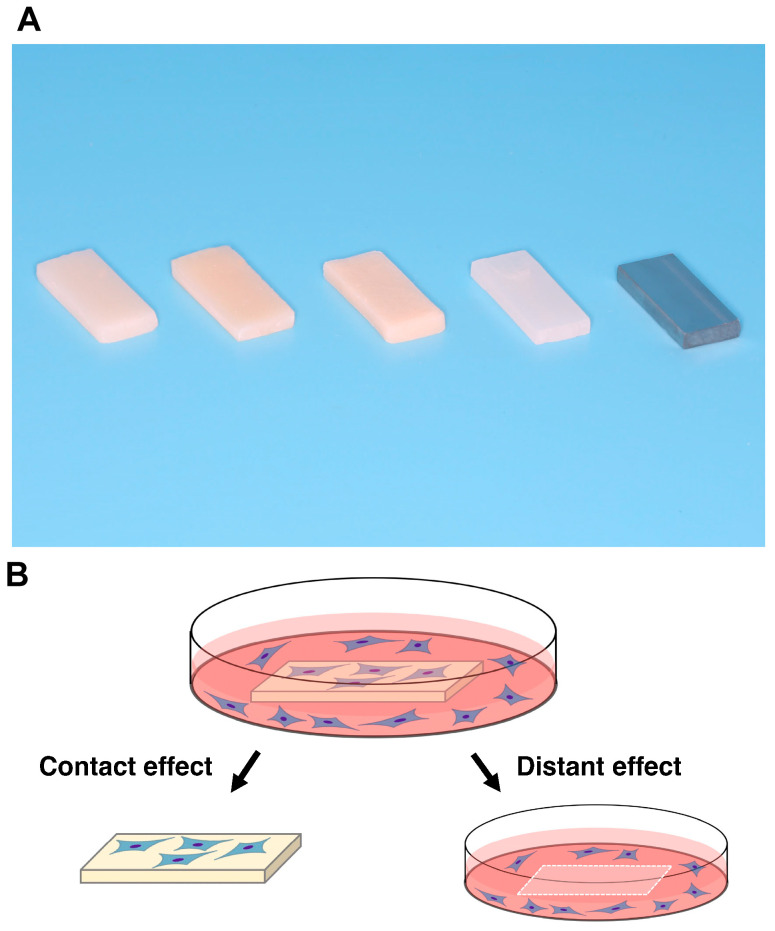
Test materials and the experimental design for counting cells. (**A**) Prepared rectangular samples (6 mm × 14 mm, 2 mm thick). a, bis-acrylic; b, composite; c, self-curing acrylic; d, milled acrylic; and e, Ti alloy. (**B**) Attached cells were counted to determine contact and proximity effects, where the contact effect was the quantification of cells attached to test materials and the proximity effect was the quantification of cells attached to the well of the culture dish (20 mm diameter) around the materials.

Five different test materials in rectangular plate form (6 mm × 14 mm, 2 mm thick) were prepared (Figure 1A, Table 1). Bis-acrylic, composite, and self-curing acrylic were prepared using standardized silicone molds prepared for each material and according to the manufacturer’s instructions. Milled acrylic plates were designed using CAD software (123D Design, Hyperdent^®^, Synergy Health, Sydney, Australia) and machined from PMMA disks with a milling machine (Versamill 5 × 200, Axsys Dental Solutions, Wixom, MI, USA). Acrylic plates were washed with a steam cleaner and disinfected with 75% ethanol. Machined Ti alloy plates were manufactured as a positive control. Surface topography was examined by scanning electron microscopy (SEM; Nova 230 Nano SEM, FEI, Hillsboro, OR, USA).

### 2.2. Cell Culture

Human gingival fibroblasts were purchased from ScienCell Research Laboratories (Carlsbad, CA, USA) and grown in a fibroblast medium supplemented with 5% fetal bovine serum (FBS), 1% fibroblast growth supplement-2, and 1% penicillin/streptomycin solution. Immortalized human bone marrow mesenchymal stromal cells (hMSCs) were purchased from Applied Biological Materials Inc. (Richmond, VC, Canada) and grown in alpha-modified Eagle’s medium (αMEM) supplemented with 15% FBS and 1% antibiotic-antimycotic solution. Cells were incubated in 95% air and 5% CO_2_ at 37 °C. The medium was changed every three days, and the cells were passaged with 0.05% trypsin-EDTA at 80% confluence. Cells at passages 5–8 were seeded at a density of 4 × 10^4^ cells/well onto each test material placed in a well (20 mm diameter) of 12-well culture plates. An osteogenic induction medium consisting of the growth medium with 100 nM dexamethasone, 10 mM sodium β-glycerophosphate, and 0.05 mM ascorbic acid was used from the time of seeding. Therefore, the cells were defined and termed as osteoblasts from the onset of seeding in the present study. The UCLA Institutional Biosafety Committee approved the study protocol (BUA-2-22-036-001).

### 2.3. Quantification of Attached and Propagated Cells

The number of attached cells was counted to determine the “contact” effect and the “proximity” effect, where the contact effect was the quantification of cells attached directly to the test materials and the proximity effect was the quantification of cells attached to the well of the culture dish around the materials (Figure 1B). Water-soluble tetrazolium salt (WST-1)-based colorimetric assays were used to quantify cell viability. Attached cells were measured two days after seeding, while propagated cells were measured four and six days after seeding. The amount of formazan product was measured at an absorbance of 450 nm using a microplate reader (Synergy H1, BioTek Instruments, Winooski, VT, USA).

### 2.4. Fluorescence Microscopy

Cell morphology was visualized four days after seeding by fluorescence microscopy (DMI6000B, Leica Microsystems, Wetzlar, Germany). Cells were dual stained with DAPI to visualize nuclei and rhodamine-phalloidin to visualize actin filaments. Cell density was quantified on each test material by counting cells visualized in the images.

### 2.5. Collagen Production

Fibroblast collagen production was quantified with a soluble collagen assay (ab241015 Soluble Collagen Assay Kit, Abcam, Cambridge, UK). Four days after seeding, cells were manually detached and collected in PBS, pelleted by centrifugation, and 0.5 M acetic acid was added to the cell pellet. Soluble collagen in acetic acid, followed by enzymatic degradation of collagen into glycine-rich oligopeptides, was quantified using a fluorogenic reagent and developer solution that selectively reacts with N-terminal glycine fragments to form a stable fluorescent complex (Ex/Em = 376/468 nm).

### 2.6. Alkaline Phosphatase (ALP) Activity

Osteoblast ALP activity was examined on day four using a colorimetry-based assay. After removing the samples from the culture wells, the wells were rinsed with double-distilled water (ddH_2_O) and treated with 250 µL p-nitrophenyl phosphate before further incubation at 37 °C for 15 min. ALP activity was evaluated as the amount of nitrophenol released through the enzyme reaction and measured at an absorbance of 405 nm using a microplate reader.

### 2.7. Compatibility Index for Fibroblasts and Osteoblasts

To quantify which cell type was more susceptible to the toxicity of each material, a compatibility index was calculated by dividing the higher number of fibroblasts or osteoblasts by the lower number of fibroblasts or osteoblasts. If the numerator was the number of fibroblasts, the material provided a more favorable, profibroblastic environment, and vice versa. The index score was calculated relative to the Ti alloy.

Using a WST-1 value for the contact effect of the self-curing acrylic on day 2 as an example, the compatibility index was expressed as (WST-1 value of fibroblasts on self-curing acrylic/WST-1 value of fibroblasts on titanium alloy)/(WST-1 value of osteoblasts on self-curing acrylic/WST-1 value of osteoblasts on titanium alloy).

### 2.8. Statistical Analysis

Results are expressed as means ± standard deviations from triplicate experiments (n = 3). The five materials were compared with a one-way analysis of variance (ANOVA) with Bonferroni post hoc correction. Two-way ANOVA was performed to evaluate differences between the test materials at varying time points. Two groups were compared using Student’s *t*-test. Any *p*-values less than 0.05 (alpha value of 0.05 and confidence level of 0.95) were deemed statistically significant.

## 3. Results

### 3.1. Surface Characteristics of the Test Materials

**Figure 2:**
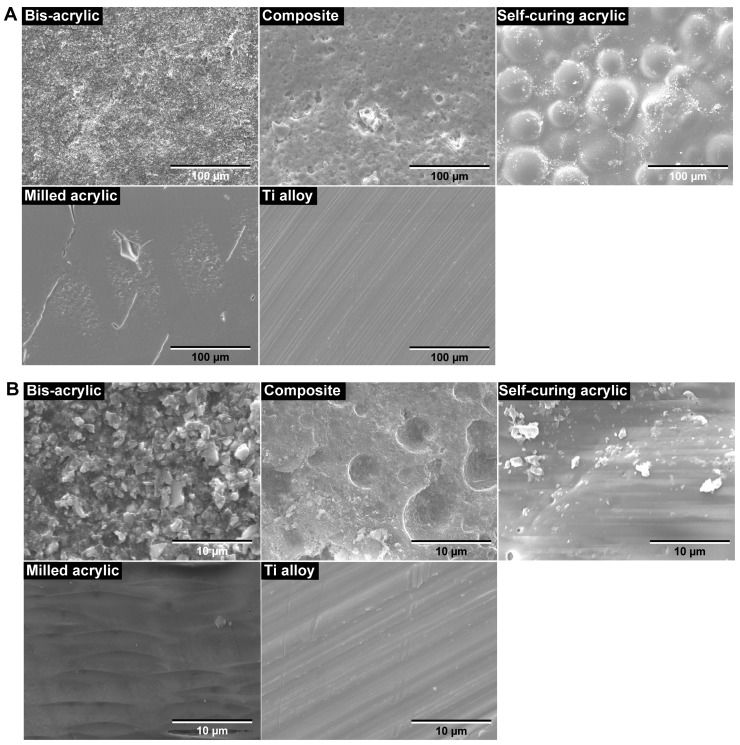
Surface topography of the test materials by scanning electron microscopy (SEM). (**A**) Low-magnification SEM images (×1000). (**B**) High-magnification SEM images (×10,000).

Scanning electron microscopy revealed that the surface morphology was highly variable between the test materials. In low-magnification images, the Ti alloy had the smoothest surface of all the test materials, showing minor machining traces only (Figure 2A). The smoothness of milled acrylic was comparable to the Ti alloy surface, apart from the presence of small scratches. Bis-acrylic had the roughest surface, while self-curing acrylic had a rough surface with spherical structures suggesting polymer particles. The composite had a rough and irregular surface at the micron level. In high magnification images, Ti alloy, milled acrylic, and self-curing acrylic had smooth surfaces (Figure 2B), bis-acrylic had a micro-rough surface, and composite had a combination of large-scale irregularities and pores on the surface.

### 3.2. Initial Attachment of Fibroblasts and Osteoblasts

To evaluate the attachment of fibroblasts and osteoblasts after seeding, we quantified the number of viable cells attached to the test materials (contact effect) and to the culture dish wells around the test materials (proximity effect) using WST-1 assays two days after seeding. Cell attachment was highly variable on different test materials.

**Figure 3:**
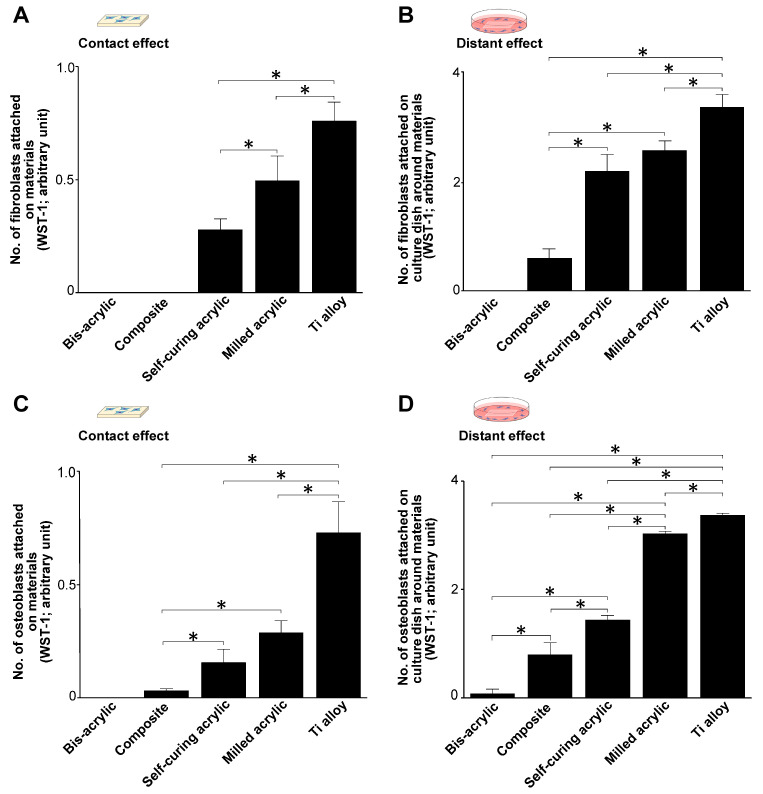
Successful attachment of fibroblasts and osteoblasts during initial culture (day 2). (**A**) The number of attached fibroblasts on test materials (contact effect). (**B**) The number of fibroblasts attached to the culture dish around the test materials (proximity effect). (**C**) The number of attached osteoblasts in contact experiments and (**D**) in proximity experiments. Data shown are mean ± SD. Significant differences are shown (one-way ANOVA with the Bonferroni post hoc test, * *p* < 0.05).

The attachment of fibroblasts and osteoblasts was greatest to Ti alloy followed by milled acrylic. Approximately twice the number of fibroblasts attached to milled acrylic than to self-curing acrylic, although this was ~30% lower than to the Ti alloy (Figure 3A). No cells attached to bis-acrylic and composite. The results of proximity experiments were similar except that some fibroblasts attached around the composite (Figure 3B).

Osteoblasts attached to milled acrylic, albeit ~50% less than to the Ti alloy. In contrast to fibroblasts, few osteoblasts attached to the composite (Figure 3C). In proximity experiments, osteoblasts attached around milled acrylic at ~90% of the level as around Ti alloy. A small number of osteoblasts also attached around bis-acrylic (Figure 3D).

### 3.3. Propagation of Fibroblasts and Osteoblasts

**Figure 4:**
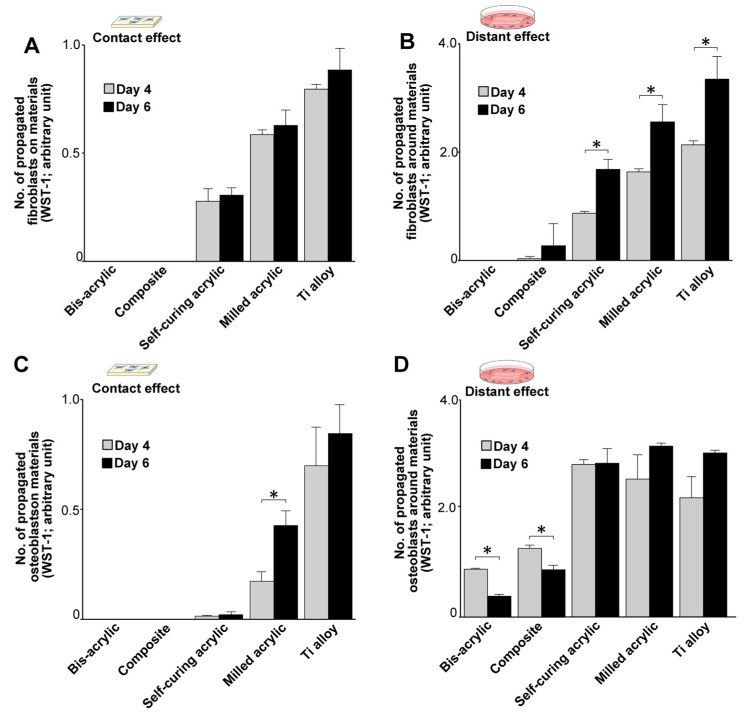
Cell propagation after initial settlement (days 4 and 6). (**A**) The number of propagated cells on test materials. (**B**) The number of propagated cells around test materials. (**C**) The number of propagated osteoblasts on test materials. (**D**) The number of propagated osteoblasts around test materials. Data shown are means ± SD (n = 3). Significant differences are shown (Student’s *t*-test, * *p* < 0.05).

We assessed the number of propagated cells on days four and six of culture. In contact experiments, the greatest propagation of fibroblasts was on Ti alloy on both days followed by milled acrylic and self-curing acrylic (Figure 4A), with slightly more fibroblasts present on day six than on day four. Proximity experiments showed a similar trend (Figure 4B), although there was a greater time-dependent increase in the number of propagated cells around Ti alloy, milled acrylic, and self-curing acrylic than in contact experiments. Proliferative activity was low around the composite.

With respect to osteoblasts, the highest propagation of osteoblasts was on Ti alloy on both days in contact experiments (Figure 4C), and there were significantly more cells on day six than on day four on milled acrylic. There was very little cell propagation on self-curing acrylic. In proximity experiments, Ti alloy, milled acrylic, and self-curing acrylic showed similar levels of cell propagation on the adjacent culture dish (Figure 4D), and there were significantly fewer cells around bis-acrylic and composite on day six than on day four.

### 3.4. Visualization of Fibroblasts and Osteoblasts

**Figure 5:**
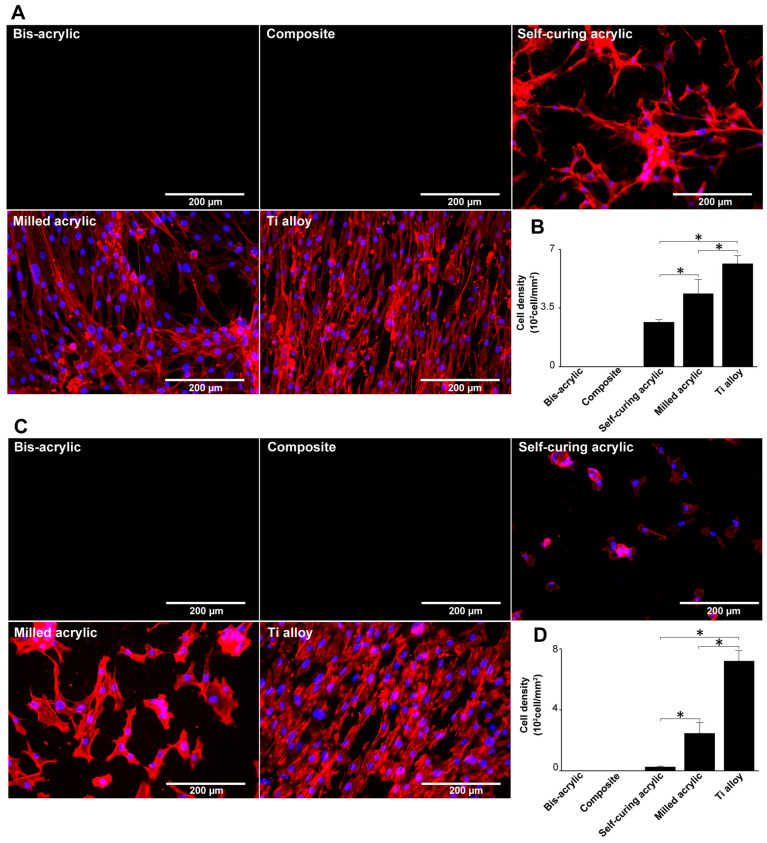
Visualization of fibroblasts and osteoblasts on test materials 4 days after seeding. (**A**) Fibroblasts were dual stained with DAPI for nuclei and rhodamine-phalloidin for actin filaments. (**B**) The number of fibroblasts in these images was counted to confirm the result in Figure 3A. (**C**) Osteoblasts stained similarly to fibroblasts. (**D**) The number of osteoblasts in these images was counted to confirm the result in Figure 3C. Data shown are means ± SD (n = 3). Significant differences are shown (one-way ANOVA with the Bonferroni post hoc test, * *p* < 0.05).

Fibroblasts and osteoblasts were visualized by dual staining with DAPI for nuclei and rhodamine-phalloidin for actin filaments. On milled acrylic and Ti alloy, propagated fibroblasts were spindle-shaped with a positively staining cytoskeleton and outline (Figure 5A). While some cells were present on the self-curing acrylic, the cells remained separated with irregular outlines. Quantification of cell density showed the highest number of fibroblasts on Ti alloy followed by milled acrylic and self-curing acrylic (Figure 5B), corroborating the WST-1 assay results in contact experiments.

Osteoblasts propagating on milled acrylic and Ti alloy were spindle shaped (Figure 5C), with some separation on the milled acrylic. Osteoblasts on self-curing acrylic were small and rounded or square. Both cell types were not observed on bis-acrylic and composite. Osteoblasts were 2.5 times denser on Ti alloy than on milled acrylic and were present at a low density on self-curing acrylic (Figure 5D). Again, the results corroborated the WST-1 assay results in contact experiments.

### 3.5. Collagen Production by Fibroblasts

**Figure 6:**
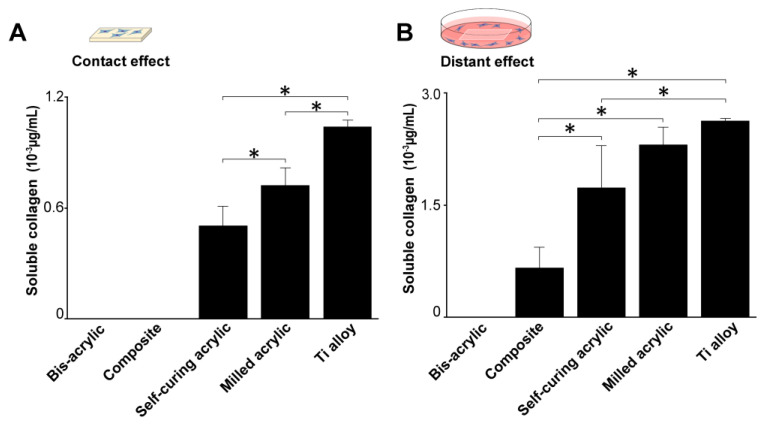
Collagen production by fibroblasts 4 days after seeding. (**A**) Collagen production on test materials and (**B**) around test materials. Data shown are means ± SD (n = 3). Significant differences are shown (one-way ANOVA with Bonferroni post hoc test, * *p* < 0.05).

Fibroblast collagen production was measured four days after seeding. In contact experiments, collagen production was greatest on the Ti alloy followed by milled acrylic and self-curing acrylic (Figure 6A). Collagen production on milled acrylic was approximately 25% lower than on the Ti alloy and there was no collagen production on the bis-acrylic and composite. In proximity experiments, collagen production around the milled acrylic was comparable to the Ti alloy (Figure 6B), while fibroblasts around the composite produced a quarter of the collagen produced around the milled acrylic. There was no collagen production around bis-acrylic.

### 3.6. ALP Activity of Osteoblasts

**Figure 7:**
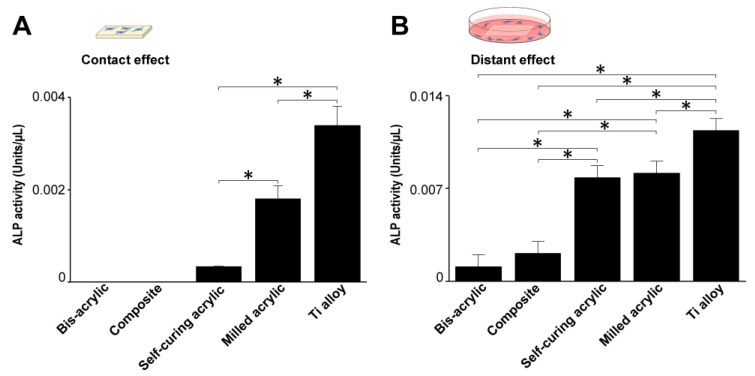
Alkaline phosphatase (ALP) activity of osteoblasts 4 days after seeding. (**A**) ALP activity on test materials and (**B**) around test materials. Data shown are means ± SD (n = 3).Significant differences are shown (one-way ANOVA with Bonferroni Post hoc test, *p < 0.05).

Osteoblast ALP activity was measured four days after seeding. In contact experiments, ALP activity was highest on the Ti alloy followed by milled acrylic (about 50% lower) (Figure 7A). Osteoblasts growing on the self-curing acrylic showed some ALP activity. In proximity experiments, the ALP activity of cells around the milled acrylic was 20% lower than those around the Ti alloy and comparable to the self-curing acrylic (Figure 7B). The ALP activity of cells around bis-acrylic and composite was 4–5 times lower than that around the milled acrylic.

### 3.7. Compatibility Index

We next assessed the favorability of the environment for fibroblasts and osteoblasts compared with Ti alloy using a compatibility index, where the higher number of fibroblasts or osteoblasts was divided by the lower number. Fibroblasts as the numerator were interpreted as the material providing a more favorable environment for fibroblasts than osteoblasts. The index score was relative to the Ti alloy.

**Figure 8:**
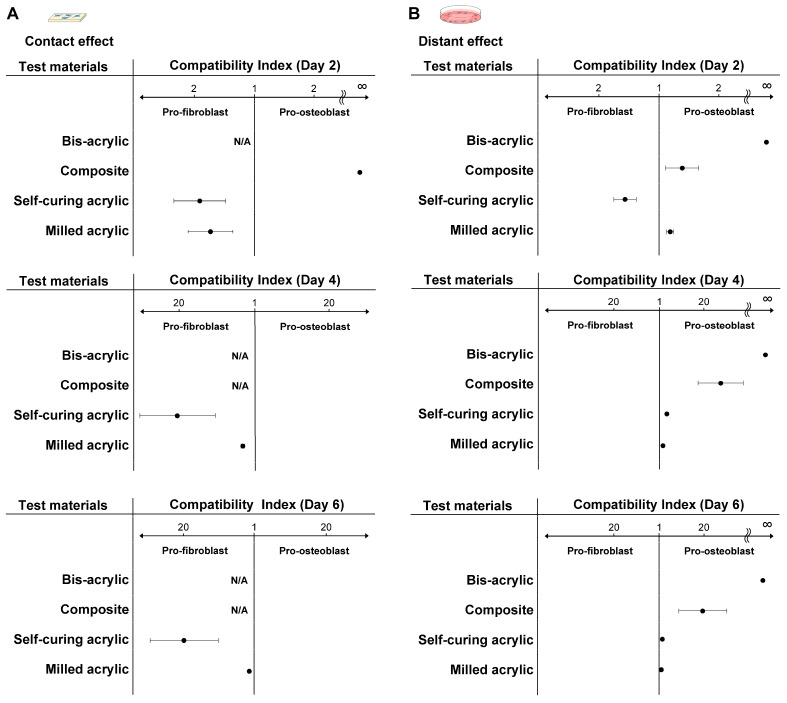
Favorable environments for fibroblasts and osteoblasts compared with Ti alloy. The compatibility index represents the higher number of fibroblasts or osteoblasts divided by the lower number. If the numerator was the number of fibroblasts, the material provided a more favorable, pro-fibroblastic environment. The index score was relative to Ti alloy. (**A**) Compatibility index related to the contact effect and (**B**) the proximity effect. Abbreviation: N/A, not applicable.

In contact experiments, self-curing acrylic and milled acrylic were more favorable for initial fibroblast attachment and propagation than osteoblasts (Figure 8A). At all time points, milled acrylic and self-curing acrylic showed higher compatibility with fibroblasts. The index score for self-curing acrylic was 20 times higher than milled acrylic on days four and six. The composite showed higher compatibility with osteoblasts with an extremely high index on day two. The index score of the milled acrylic approached one in a time-dependent manner.

With respect to proximity experiments, bis-acrylic and composite showed higher compatibility with osteoblasts than fibroblasts at all time points (Figure 8B). The bis-acrylic index score was unlimited because no fibroblasts attached to the wells around the material. In contrast to contact experiments, self-curing acrylic showed higher biocompatibility with osteoblasts on day four, and the index scores of the self-curing and milled acrylic were nearly one on day six.

## 4. Discussion

Here we comprehensively assessed the biocompatibility of implant provisional restoration materials with human gingival fibroblasts and osteoblasts by evaluating cellular behavior and function on and around the materials. This allowed us to determine whether the biocompatibility varied for different cell types and whether it differed when cells were in direct contact or adjacent to the potentially cytotoxic materials.

This study implied that implant provisional restoration materials may adversely affect soft tissue healing and osseointegration depending on the material. In culture experiments, fibroblasts and osteoblasts can either adhere to materials or around the materials, with those that do not attach undergoing cell death. Therefore, quantifying the number of cells attaching to either the test materials or the culture wells represents an indirect measurement of cell survival in the face of material cytotoxicity. Here, we tested four representative provisional restoration materials and Ti alloy, the latter chosen as a positive control because it is known to be bioinert and widely used for temporary abutments [35,36,37,38,39,40]. The number of cells attaching to a test material was regarded as a contact effect, while the number of cells attaching to the well around the test material was regarded as a proximity effect. We found that initial fibroblast and osteoblast settlement/attachment and subsequent propagation varied considerably ranging from lethal and tolerant to inert, depending on the material tested. As expected, the highest number of cells attached and propagated on the Ti alloy at all time points. Unexpectedly, osteoblasts, but not fibroblasts, attached to the composite. Compared with Ti alloy, fewer osteoblasts attached to the self-curing acrylic and milled acrylic than fibroblasts. However, the milled acrylic showed close cytocompatibility to the Ti alloy and the self-curing acrylic was significantly less cytotoxic than bis-acrylic and composite. As expected, attachment and proliferation improved slightly in proximity experiments, suggesting that there was less of a chemical effect exerted on cells in close proximity to the materials. Surprisingly, only a few osteoblasts attached around bis-acrylic. Of clinical note, the fibroblasts and osteoblasts had differing susceptibilities to the cytotoxic effects of the tested materials.

Some studies have shown that the cytotoxicity of materials depends on their constituents including monomers, polymerization initiators, and filler particles [32,41,42,43]. Unreacted monomers have critical cellular effects [16,30]. Bis-GMA and UDMA are the main ingredients of the composite, and bis-GMA is eluted at higher concentrations than UDMA [44]. Both cause DNA strand breaks in fibroblasts, which would account for the observed cytotoxicity [45]. Our results suggest that bis-GMA and UDMA are more toxic than MMA, while UDMA is less toxic than bis-GMA, especially to fibroblasts. Monomers do not appear to be eluted from milled acrylic [46], and our results confirm that milled acrylic is more cytocompatible than self-curing acrylic, most likely due to the lower residual monomer composition.

Benzoyl peroxide (BPO) and camphorquinone (CQ) are major polymerization initiators that can also compromise cell viability. BPO is an initiator for the self-curing acrylic resin that is broken down during polymerization to release radicals that injure adjacent cells [17,23,24,28,31]. CQ is a well-known photoinitiator for composite resins that promotes polymerization by generating free radicals with an amine as a coinitiator [47]. Our results show that self-curing acrylic is less cytotoxic than composite, suggesting that BPO is likely to be less cytotoxic than CQ.

Collagen production was generally proportional to the number of cells, but the production of collagen around the self-curing acrylic was disproportionately higher than around the other materials. This means that self-curing acrylic is less deleterious to fibroblast function. In addition, the milled acrylic had no toxic effect on collagen production due to the much lower residual monomer composition than self-curing acrylic.

Osteoblasts growing on the Ti alloy had the highest ALP activity, followed by those on milled acrylic. The ALP activity of cells growing around the self-curing acrylic was similar to that around milled acrylic, so self-curing acrylic may have little deleterious effect on osteoblast function. Despite some osteoblasts attaching and propagating on the composite, there was no detectable ALP activity. There was also a time-dependent decrease in the number of osteoblasts growing on bis-acrylic and composite, highlighting persistent toxicity resulting in compromised osteoblast function for both materials.

It is known that the surface topography of biomaterials influences the initial cellular behavior [48,49,50,51,52]. In theory, the smoother the surface, the better the cell attachment and proliferation [12,51,53,54,55,56,57,58]. Although the primary focus of this study was the chemical effect of materials on cellular activity, qualitative analysis of the surface morphology by SEM provided some useful insights into the cell-material interactions. For example, cell attachment and proliferation were higher on materials with relatively smooth surfaces (self-curing acrylic, milled acrylic, and Ti alloy), consistent with the hypothesis that smooth surfaces promote attachment and proliferation. These trends were especially prominent for osteoblasts. Based on these insights and hypothesis, future studies are required to quantitatively assess the surface morphology of the test materials including the average roughness and the peak-to-valley roughness to explore the contribution of topography/roughness factors in determining the biocompatibility of implant provisional materials.

The hybrid assessment of contact and proximity effects was designed to mimic the local environment of peri-implant tissue, i.e., the connective tissue and alveolar bone, both of which are exposed directly and indirectly to provisional materials. The optimal environment for fibroblasts and osteoblasts was indicated by our compatibility index, which varied according to the assay. Self-curing and milled acrylic were pro-fibroblastic at all time points, with self-curing acrylic showing the highest index and milled acrylic approaching a value of one due to later osteoblastic plateauing.

Conversely, results were different in proximity experiments, with bis-acrylic and composite indices pro-osteoblastic. Milled acrylic was slightly pro-osteoblastic at all time points. In another study examining the cytotoxicity of different surface treatments of composite, there was a trend towards a pro-osteoblastic phenotype [59], consistent with our results. We separately evaluated the contact and proximity effects of each material for fibroblasts and osteoblasts and provided robust, fundamental data, which warrants further studies such as in vitro mechanistic studies and animal studies.

## 5. Conclusions

Here we systematically examined the biocompatibility of various implant provisional restoration materials on fibroblasts and osteoblasts in direct contact with, or close proximity to, the materials. The number of fibroblasts and osteoblasts surviving, and attaching to and around the materials, varied considerably depending on the material, with milled acrylic the most biocompatible and similar to titanium alloy, followed by the self-curing acrylic; however, little to no attachment on or around the bis-acrylic and composite materials. Milled and self-curing acrylics similarly favored subsequent cellular functions such as collagen production in fibroblasts and alkaline phosphatase activity in osteoblasts. No functional phenotype was detected in fibroblasts and osteoblasts cultured with bis-acrylic and composite. Fibroblasts were more resistant to cytotoxicity induced by most materials in direct contact, however, osteoblasts were more resistant when the materials were in close proximity. Thus, there was a wide variation in the cytotoxicity of implant provisional materials ranging from lethal and tolerant to near inert. This cytotoxicity may be received differently between the different cell types and depending on their physical interrelationships. These results establish a foundation for understanding and selecting materials during implant provisional restoration and warrant future in vivo studies to further explore their biocompatibility at the tissue level.

## Figures and Tables

**Table 1:** Materials used in this study.

Materials	Product Name (Manufacturer)	Principal Ingredients
Bis-acrylic	Integrity^®^ Multi Cure Temporary Crown and Bridge Material (Dentsply Sirona Inc.)	Acrylates and methacrylates (bis- and multifunctional) Barium boro alumino silicate glass
Composite	Aelite™ Aesthetic Enamel (BISCO Inc.)	Bis-GMA, UDMA
Self-curing acrylic	JET™ Tooth Shade (Lang Dental Manufacturing Company Inc.)	Liquid: MMA Powder: 2-Propenoic acid, 2-methyl-, methyl ester, homopolymer
Milled acrylic	Vivid PMMA Disc (Pearson™ Dental Supply Co.)	PMMA
Ti alloy	−	Ti-6Al-4V (Grade 5)

Abbreviations: UDMA, urethane dimethacrylate; Bis-GMA, bisphenol A glycidyl methacrylate; MMA, methyl methacrylate; PMMA, poly (methyl methacrylate).

## Data Availability

The data presented in this study are available on request from the corresponding author.
